# Mapping Atrial Tachycardia Post‐Pulmonary Vein Isolation Using the PulseSelect Catheter and Ensite NavX

**DOI:** 10.1002/joa3.70206

**Published:** 2025-10-22

**Authors:** Takayuki Okamoto, Takashi Okajima, Shinji Ishikawa, Yusuke Uemura

**Affiliations:** ^1^ Department of Cardiology Anjo Kosei Hospital Anjo Japan

**Keywords:** atrial tachycardia, peak frequency, pulmonary vein isolation, pulsed field ablation, three‐dimensional electroanatomical mapping

## Abstract

The nine 3‐mm electrodes, 3.75‐mm fixed interelectrode space, and soft and slightly tilted shape of the PulseSelect catheter enables creating high‐quality 3D electroanatomical mapping. Combined with an Ensite NavX 3D mapping system, it may be a superior option for managing atrial tachycardia after pulmonary vein isolation with pulsed field ablation.

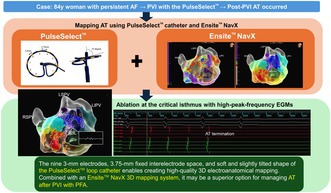

Macro‐reentrant atrial tachycardias (ATs) frequently occur after pulmonary vein isolation (PVI); accurately identifying the tachycardia circuit and revealing the critical isthmus remain clinical challenges. High‐resolution three‐dimensional (3D) electroanatomical mapping systems, in combination with multielectrode high‐density mapping catheters, have facilitated accurate diagnosis of AT circuits and resulted in high success rates of catheter ablation [1]. Pulsed‐field ablation (PFA), based on electroporation, has emerged as a safe and effective alternative to thermal ablation of PVI [2]. Although PFA catheters have multiple electrodes that allow the acquisition of electrogram information, their clinical utility and accuracy in mapping ATs following PVI have not been fully elucidated. Herein, we present a case of AT following PVI with PFA, which was successfully treated based on the 3D electroanatomical mapping findings obtained with a PulseSelect loop PFA catheter (Medtronic, Minneapolis, MN) combined with an EnSite NavX 3D mapping system (Abbott, Chicago, IL).

An 84‐year‐old woman was referred with tachycardia and palpitations. Electrocardiogram (EGM) revealed atrial fibrillation (AF), and echocardiography showed a normal left ventricular contraction and moderate left atrial enlargement. After obtaining written informed consent, catheter ablation was performed under deep sedation. PVI was performed using the PulseSelect PFA system combined with the Ensite NavX 3D mapping system. All pulmonary veins (PVs) were successfully isolated under a J‐tipped guidewire navigation; however, the left and right superior PVI lines were connected to form a roof block line. Subsequently, sustained AT with a tachycardia cycle length of 266 ms was induced. AT mapping was performed using the PulseSelect loop catheter (Figure 1) integrated with the EnSite NavX system without changing the catheter. Bipolar near‐field detection was selected with the reference electrode placed in the coronary sinus. To avoid the risk of the knotting phenomenon of the PulseSelect catheter [3], we kept the guidewire tip approximately 2 cm away from the catheter tip, avoided pushing and pulling the wire, and carefully manipulated the catheter without unnecessary rotations or stretches during mapping. More than 17 000 points were acquired within several minutes; approximately 3000 points demonstrating dense coverage of the entire surface of the left atrium were used in the final map (Figure 2). The voltage map showed low‐voltage areas on the anterior wall of the left atrium. The activation map clearly demonstrated a re‐entrant AT circuit with a critical isthmus located at the anterior wall of the left atrium. Entrainment pacing was performed at a site within the suspected circuit to confirm the diagnosis. Moreover, the peak‐frequency map demonstrated high‐peak‐frequency EGMs at 192 Hz, as shown in the activation map using the emphasis mapping technology (Figure 3). Since the target site was not around the PVs, we selected a 4.0‐mm irrigated flexible‐tip radiofrequency ablation catheter (TactiFlex SE, Abbott, Chicago, IL); AT was terminated within 2 s after the initial radiofrequency application (Figure 4). Post‐ablation pacing did not induce any atrial arrhythmias. Moreover, no recurrence was observed during the 6‐month follow‐up period.

**FIGURE 1:**
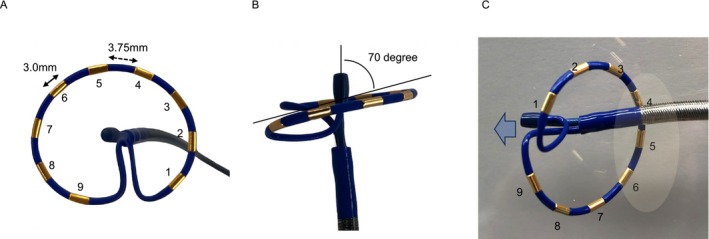
Images showing the front (A) and lateral (B) view of the PulseSelect loop catheter. The catheter has nine 3‐mm long electrodes with a fixed interelectrode spacing of 3.75 mm and a soft and slightly tilted shape. Stable electrode contact was observed, especially around electrode no. 5 when the catheter was pressed against the wall and advanced (C). The numbers indicate the electrode numbers.

**FIGURE 2:**
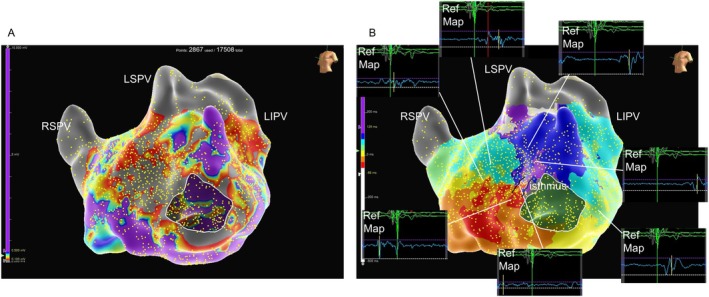
Voltage (A) and activation (B) maps obtained during AT using the with PulseSelect loop catheter combined with the EnSite NavX system. A total of 2888 points (yellow dots) were selected from 17 596 acquired points, demonstrating dense coverage on the entire surface of the left atrium. The maps clearly show low‐voltage areas and the critical isthmus of the reentrant AT circuit on the anterior wall of the left atrium. Accurate annotations are observed even for small potentials within the low‐voltage areas. AT, atrial tachycardia; LIPV, left inferior pulmonary vein; LSPV, left superior pulmonary vein; RSPV, right superior pulmonary vein.

**FIGURE 3:**
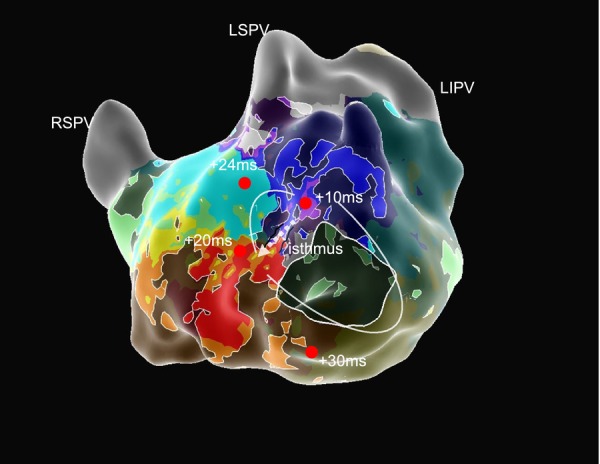
Emphasize map highlights areas with a peak frequency greater than 190 Hz. The highlighted area corresponds to the isthmus of the AT. The schema image illustrates the entire suspected AT circuit, with the white dotted arrow indicating the activation pathway. The red tags show the site where entrainment pacing was performed, and the difference between the post pacing interval (PPI) and tachycardia cycle length (TCL) is indicated. LIPV, left inferior pulmonary vein; LSPV, left superior pulmonary vein; ms, milliseconds; RSPV, right superior pulmonary vein.

**FIGURE 4:**
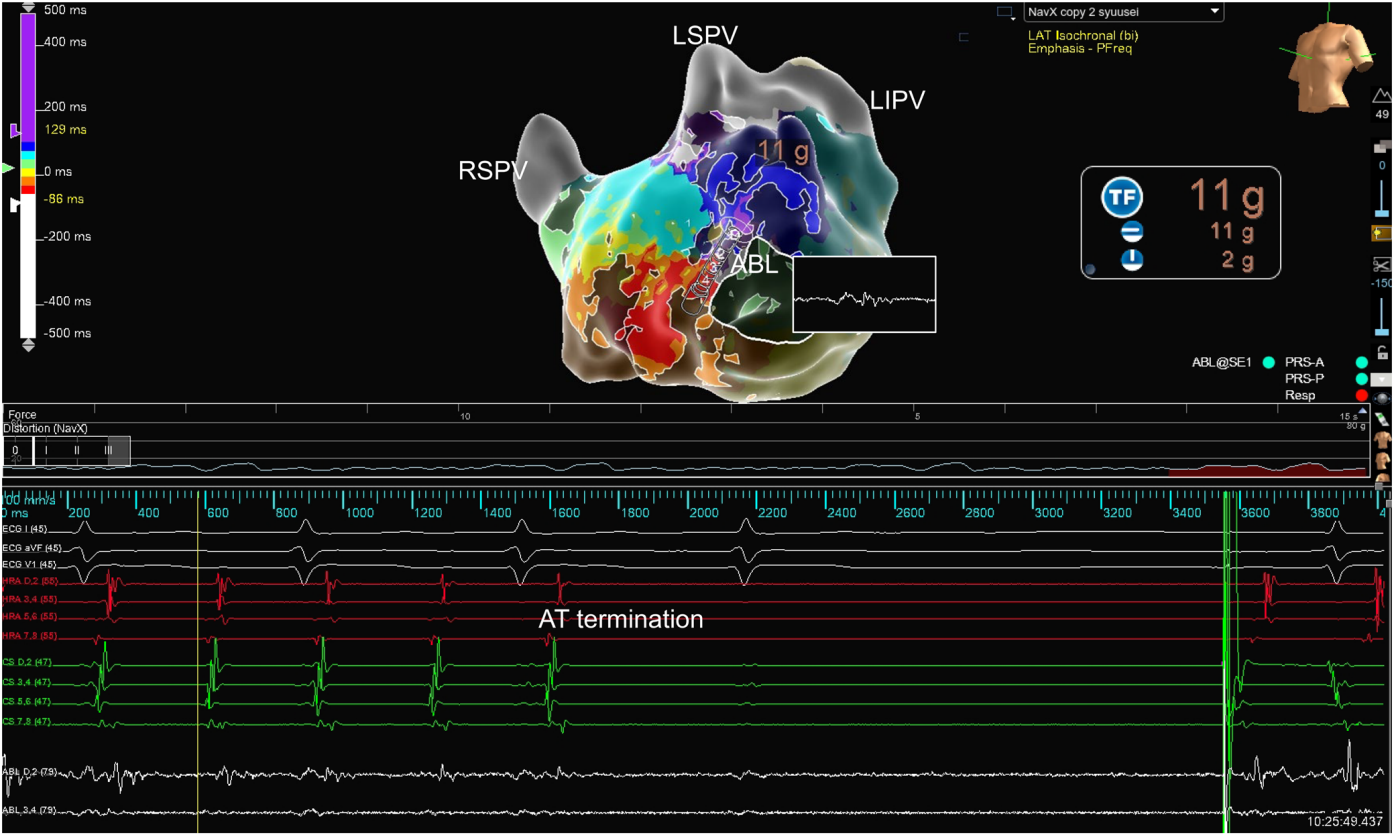
The AT was terminated within 2 s after the initial radiofrequency application targeting the critical isthmus. A long‐duration fragmented potential has been recorded on the ablation catheter at the target site. ABL, ablation catheter; AT, atrial tachycardia; LIPV, left inferior pulmonary vein; LSPV, left superior pulmonary vein; RSPV, right superior pulmonary vein.

The PulseSelect loop catheter, characterized by its nine 3‐mm long electrodes and fixed interelectrode space of 3.75 mm, may enable the acquisition of higher quality electrical signals compared with other representative PFA catheters. For instance, the Farawave (Boston Scientific, Marlborough, MA) catheter has four 5‐mm electrodes on each pentaspline; however, electrical signals can only be acquired from the third electrode on each spline. Moreover, the soft and slightly tilted shape of the PulseSelect catheter facilitates stability and enables contact with the entire surface of the left atrium. Due to these features, the PulseSelect catheter possibly creates higher‐quality 3D electroanatomical mapping among the currently available PFA catheters. Although PFA has a better safety profile compared with thermal ablation, the risk of air embolism remains a concern. Owing to the use of large‐diameter sheaths, minimizing the number of catheter reinsertions may reduce the risk of air entrapment. Additionally, in resource‐limited settings, the use of a single catheter for both mapping and ablation may reduce medical costs and provide a potentially cost‐effective alternative to the use of additional multielectrode mapping catheters.

High‐frequency EGMs can be observed at the isthmus site during AT mapping using the PulseSelect catheter in combination with the EnSite NavX. The clinical utility of identifying high peak frequency (PF) sites within low‐voltage zones using the Adviser HD Grid mapping catheter (Abbott, Chicago, IL) has been reported in the mapping of complex tachyarrhythmias [4]. Although the optimal cutoff value of the PF has been reported to range between 220 and 400 Hz, the PF at the isthmus site in this case was 192 Hz. Generally, the frequency of EGMs decreases with increasing electrode size. The HD Grid mapping catheter has a smaller electrode length (1 mm) compared with the PulseSelect catheter (3 mm), which may be associated with the relatively low PF observed at the critical isthmus site.

Whether PFA or point‐by‐point radiofrequency ablation (RFA) could be more effective in treating AT after PVI remains unclear. A recent matched‐pair study comparing patients with post‐ablation ATs treated with either PFA or RFA has demonstrated comparable acute efficacy and safety. However, PFA was associated with a higher long‐term arrhythmia recurrence rate and frequently exhibited reconnection of initially ablated extra‐PV lesions [5]. Since the current PFA catheters are specifically designed for PVI, making durable transmural lesions in extra‐PV areas might be difficult. In contrast, contact force‐guided RFA with impedance drop monitoring could help achieve durable focal transmural lesions. In the present case, because the target ablation site was precisely identified using 3D mapping findings, RFA was selected and resulted in successful termination of AT by the first ablation without any complications.

In conclusion, this is the first reported case of post‐PVI AT successfully treated with 3D electroanatomical mapping with a PulseSelect loop PFA catheter combined with an Ensite NavX 3D mapping system. High‐frequency EGMs identified by the peak frequency map might indicate a critical isthmus. This approach might be a safe and cost‐effective option for managing AT after PVI with PFA. To confirm its broader applicability, specifically for more complex tachycardia circuits, further experience and validation in larger cohorts will be required.

## Disclosure

Permission to Reproduce Material From Other Sources: Available upon reasonable request.

## Ethics Statement

Approval was obtained from the local ethics committee.

## Consent

The authors obtained informed consent from the patients.

## Conflicts of Interest

The authors declare no conflicts to interests.

## Data Availability

The data that support the findings of this study are available on request from the corresponding author. The data are not publicly available due to privacy or ethical restrictions.

## References

[1] D. G. Laţcu , S. S. Bun , F. Viera , et al., “Selection of Critical Isthmus in Scar‐Related Atrial Tachycardia Using a New Automated Ultrahigh Resolution Mapping System,” Circulation. Arrhythmia and Electrophysiology 10 (2017): e004510, 10.1161/CIRCEP.116.004510. 28039280

[2] K. Kuroki and H. Tada , “Catheter Ablation Using Pulsed‐Field Energy: Advantages and Limitations Compared With Conventional Energy,” Journal of Arrhythmia 41 (2025): e70011, 10.1002/joa3.70011. 39906095 PMC11792576

[3] S. Nishiuchi , Y. Matsuo , S. Yoshimura , et al., “Fixed Knotting of Looped Pulsed Field Ablation Catheter due to Tip Inversion Without Guidewire Retraction,” JACC Clinical Electrophysiology 11 (2025): 865–867. 39918457 10.1016/j.jacep.2024.11.022

[4] J. Mayer , J. Al‐Sheikhli , M. Niespialowska‐Steuden , et al., “Detailed Analysis of Electrogram Peak Frequency to Guide Ventricular Tachycardia Substrate Mapping,” Europace 26 (2024): euae253, 10.1093/europace/euae253. 39343730 PMC11481296

[5] M. A. Gunawardene , T. Harloff , M. Jularic , et al., “Contemporary Catheter Ablation of Complex Atrial Tachycardias After Prior Atrial Fibrillation Ablation: Pulsed Field vs. Radiofrequency Current Energy Ablation Guided by High‐Density Mapping,” Europace 26 (2024): euae072, 10.1093/europace/euae072. 38513110 PMC11034699

